# A case report of infanticide in rural Nepal: Sociocultural perspectives and forensic considerations

**DOI:** 10.1002/ccr3.8078

**Published:** 2023-10-17

**Authors:** Lokaratna Gyawali, Alok Atreya, Preza Kuinkel, Rabins Sanyal, Abhishek Shah

**Affiliations:** ^1^ Rolpa District Hospital Reugha Liwang Nepal; ^2^ Department of Forensic Medicine Lumbini Medical College Palpa Nepal; ^3^ Lumbini Medical College Palpa Nepal

**Keywords:** contraceptive agents, extramarital relations, infanticide, umbilical cord

## Abstract

This case highlights the complex interplay of mental health, stigma, and lack of contraceptive access underlying tragic instances of infanticide. Comprehensive medicolegal investigation paired with cross‐sector efforts to expand reproductive services and transform cultural attitudes is crucial to protect vulnerable women and children.

## 1 INTRODUCTION

Infanticide is a global issue that affects many countries, often linked to gender discrimination, devaluation of female children, and cultural practices.^1^ Factors such as male preference, poverty, access to health care, and cultural practices contribute to the issue.^1^

Infanticide is linked to factors such as illegitimate births, unfaithful partners, adultery, and infidelity.^2^ Illegitimate births are undesirable in some cultures, leading to the killing of infants born out of wedlock.^3^ Unfaithful partners can also contribute to infanticide, as they can be seen as a violation of family honor and lead to infant's death.^2^ Adultery and infidelity can also contribute to social and cultural factors that lead to infanticide, such as social stigma and dishonor for the family.^2^, ^3^ Addressing these factors requires a multifaceted approach that considers the cultural, social, and economic context of Nepal. Infanticide is a tragic crime that transcends geographical boundaries, and this case from rural Nepal exemplifies the complexities involved in understanding and addressing this heinous act. In this case report, we present an in‐depth analysis of the circumstances surrounding the infanticide, focusing on the woman's mental state, the lack of access to contraceptives, and the cultural factors driving her actions. The purpose of this report is to shed light on the challenges faced by women in rural communities and to increase awareness and discussion of the sociocultural factors that can contribute to infanticide, such as attitudes towards adultery, stigma, and access to contraception.

## 2 CASE PRESENTATION

When police received information on the buried body, they visited the crime scene around noon in a hilly rural village in western Nepal. The body was first discovered by playing children. Upon digging and removing the soil, reddish‐colored women's pants were unwrapped, revealing a dead male fetus in a prone position. A police inquest was conducted, and the body was subjected to an autopsy.

**FIGURE 1:**
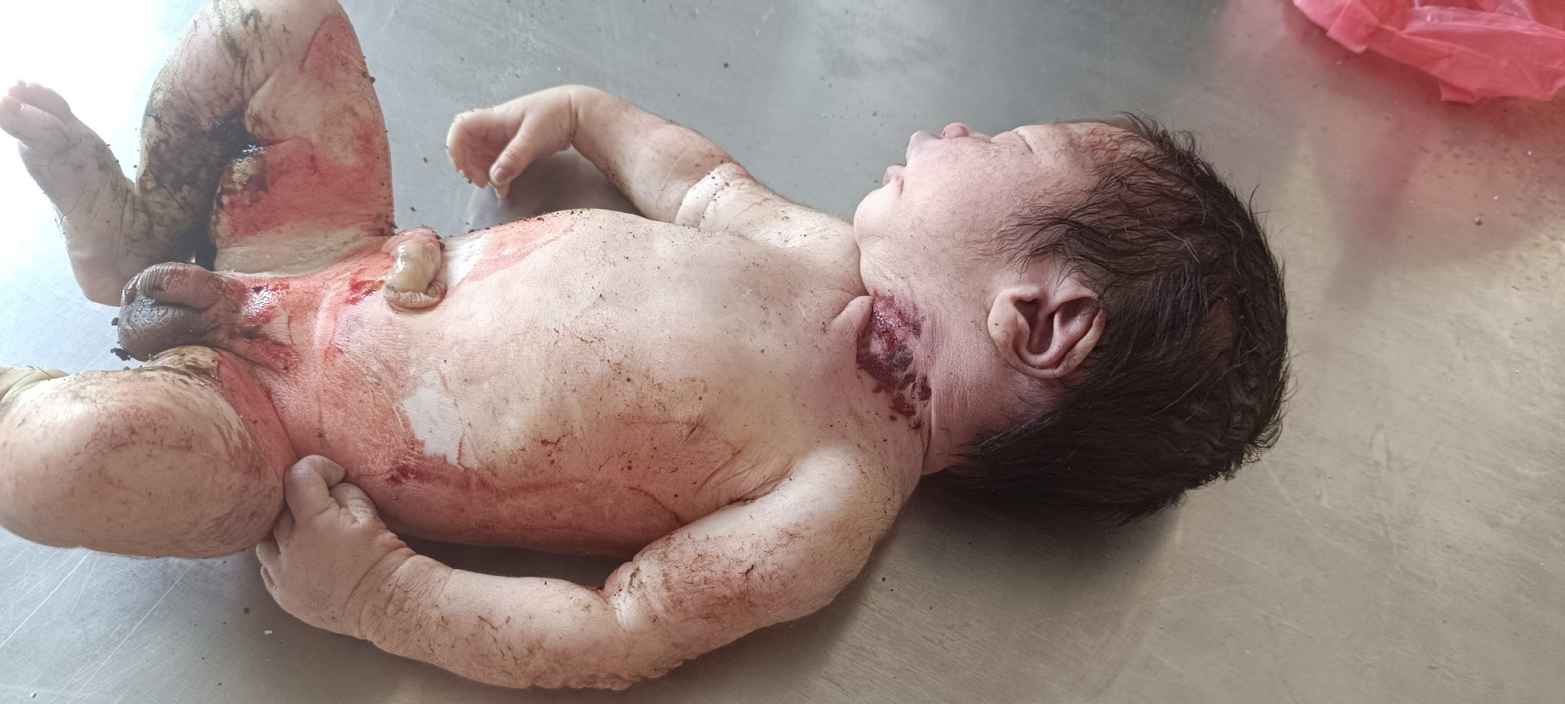
External examination of the baby revealed blood and mud smears, along with meconium stains on the perineal region and lower extremities.

**FIGURE 2:**
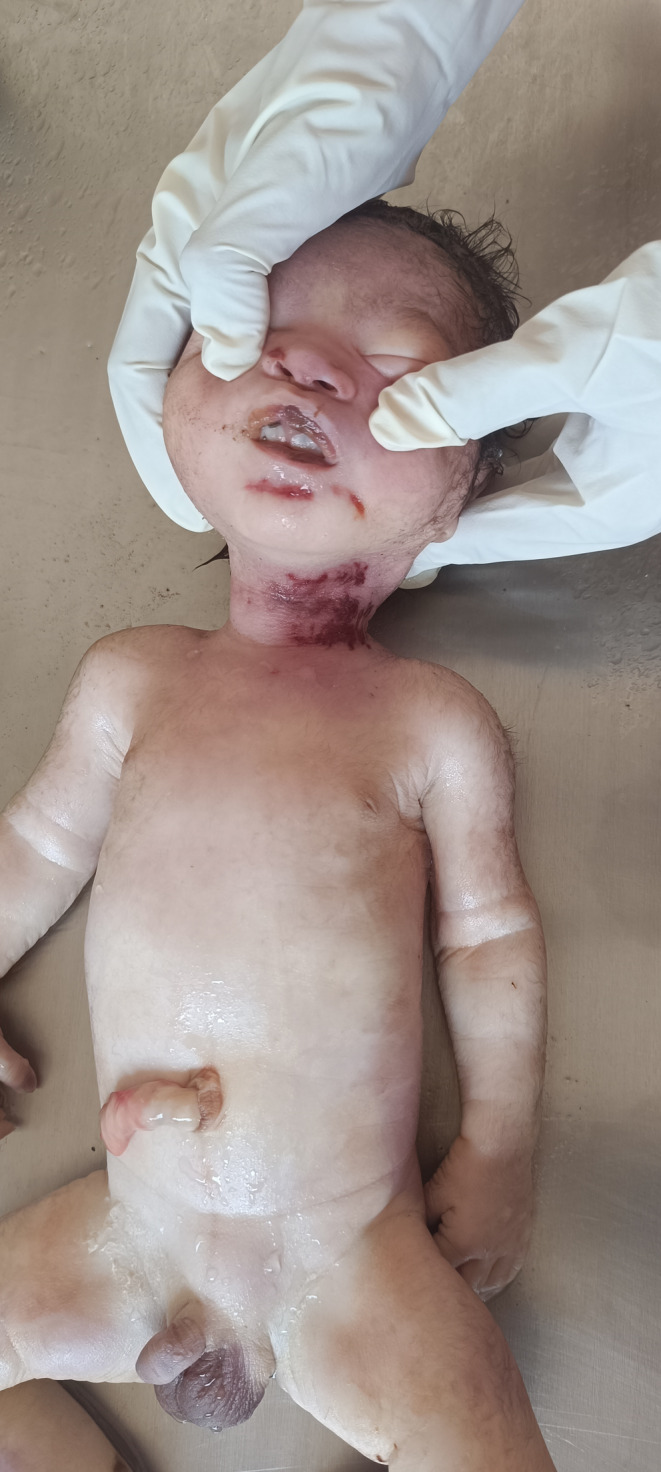
Postmortem observation of the deceased revealed bluish discoloration of the lips with a lacerated frenulum, along with three linear contusions on the left side of the neck, as well as an interrupted linear contusion on the chin.

During the external examination, the baby was found smeared in blood and mud. Meconium stains were present on the perineal region and the lower extremities (Figure 1). The head was covered in hair and the face was congested, as were the distal lower extremities. The body was cleaned with tap water and the length was measured with a measuring tape. The crown‐rump length was 32 cm, and the total body length 50 cm. The umbilical cord was severed, the edges of the free end were reddish, and the margins irregular. The testes had descended into the scrotum. The nails on both upper limbs projected beyond the fingertips. Bluish discoloration of the lips with a lacerated frenulum was evident on the deceased. There were three linear contusions, measuring 3–4 cm in length and 1 cm in width, on the left side of the neck and a 4 × 0.3 cm linear interrupted contusion on the chin (Figure 2). No gross congenital anomalies were noted.

On opening the cavity, the diaphragm was found to be at the seventh rib space. The lungs were expanded and filled the chest cavity. The cut sections of the lungs floated in water. Ossification centers of the distal epiphysis of the femur were present. The cause of death was opined to be a combined act of throttling and smothering.

Preliminary police investigation found that a women, whose husband was working as a migrant laborer in a Middle Eastern country, had an extramarital affair with a man from the same neighborhood. This resulted in her becoming pregnant. Allegedly due to social stigma, she murdered her newborn and buried it in the backyard. We could not obtain any information from the suspected woman because she was in police custody.

## 3 DISCUSSION

Cultural norms and attitudes toward adultery in rural Nepalese communities are influenced by factors such as gender roles, sociocultural norms, and traditional practices.^4^ Male‐centric sociocultural norms and practices in Nepal may contribute to attitudes toward adultery, as men have more privilege and control over relationships.^4^ Violence and silence are also prevalent, leading to underreporting of violence against women and creating an environment where women feel pressured to remain silent about infidelity.^4^ Traditional cultural practices, reflecting community values and beliefs, can also influence attitudes toward adultery, emphasizing the importance of fidelity and loyalty within relationships, leading to negative attitudes toward infidelity.^5^

Infidelity can occur in both happy and troubled relationships, and the reasons for infidelity are complex and varied.^6^ Factors contributing to infidelity include lack of affection, loss of fondness, weak commitment, breakdown of communication about emotional and relationship needs, low self‐esteem, and physical and mental health issues.^6^, ^7^

Infidelity and pregnancy are related, but not all cases of infidelity result in pregnancy. The passion of an affair can make birth control less effective, and when it results in pregnancy, it can cause lasting damage to both partners.^8^ The causes of infidelity are complex and varied, with most affairs arising from relational dissatisfaction and personal dissatisfaction.^8^ Addressing the underlying issues that lead to infidelity is crucial to prevent future infidelity.

Rural Nepal faces challenges in family planning and contraceptive accessibility due to inequalities, barriers, and the need for improved messaging and resources. Inequalities are exacerbated by lower levels of education and socioeconomic status, and rural areas often have lower availability and use of family planning methods.^9^

Men in Nepal may not use condoms due to various reasons, including sociocultural context, limited access to sexual health services, perceived stigma and shame, and limited contraceptive options.^10^ Limited access to sexual health services, such as condoms, can also hinder condom use.^11^ Perceived stigma and shame can also prevent men from using condoms, even when they are aware of the importance of safe sex practices.^10^ Additionally, limited contraceptive options, such as condoms, pills, and injectables, can restrict availability, further reducing the prevention of unintended pregnancies in Nepal.^12^ Addressing these barriers through increased access to sexual health education, reducing stigma around condom use, and expanding availability of contraceptive methods could help increase condom use and reduce unintended pregnancies in Nepal.

The impact of a husband working abroad as a migrant laborer on a woman's mental well‐being, and emotional state has been studied in Nepal.^13^ Psychiatric morbidities, such as mood disorder, anxiety, and stress‐related disorders, may be affected.^13^ Increased communication and support between migrant husbands and their wives can decrease the risk of common mental disorders and improve mental well‐being.^13^ However, Nepal is poorly equipped to treat women suffering from depression and anxiety when their husbands work abroad, with limited mental health services and resources in rural areas.^14^

Comprehensive sexual education and reproductive health programs are crucial for preventing unwanted pregnancies and infanticide. These programs educate children and adolescents on sexual behavior, contraception, sexually transmitted infections (STIs), and reproductive rights, enabling informed decisions.^15^ They reduce stigma, promote healthy attitudes, and ensure access to resources like contraception and STI testing, enabling young people to protect their sexual health and prevent unwanted pregnancies.^15^

Nepal has made significant progress in reforming its reproductive health laws and policies, including abortion.^16^ The abortion law was reformed in 2002 to ensure safe motherhood and women's rights.^16^ Nepal's experience in implementing legal abortion has served as a model for the rapid expansion of high‐quality care.^17^ However, harmful practices like infanticide continue to pervade Nepal, with enforcement challenges in rural areas due to social norms, underreporting of violence against women, and lack of faith in the justice system.^18^ Furthermore, inequalities in access to modern family planning methods among married women of reproductive age in rural Nepal are documented, with rural women often having lower education and socioeconomic status, which can reduce access to family planning and decision‐making.^9^

To reduce infanticide in Nepal, amendments to the legal system could strengthen existing laws, improve access to reproductive health services, increase awareness and education, and support vulnerable women. These measures could prevent unwanted pregnancies and protect vulnerable women in rural areas.^19^

The Nepalese judicial system is strict and would punish the culprit, who in this case is a woman. Society would label her an infidel and stigmatize her for being involved in an adulterous relationship.^20^ However, the man who had unlawful contact with the woman and impregnated her would not face legal consequences. What appears to be a crime conceals a vicious cycle of victimization and re‐victimization.^20^ The law should be amended to provide a rational interpretation in circumstances of suspected infanticide in which the man should also be brought under legal procedure and punished equally.^20^

## 4 CONCLUSIONS

This case study emphasizes the importance of a comprehensive medicolegal investigation, including forensic pathology, clinical history, and psychosocial assessment, in suspected infanticide cases. It also highlights the impact of sociocultural attitudes and stigma on unsafe contraceptive practices and extreme actions like infanticide. The mental health impacts of separation from migrant worker husbands on women's emotional well‐being and postpartum depression are significant. Access to contraceptive resources, reproductive health services, and mental health support is crucial, especially in rural areas. In‐depth case analyses help identify systemic gaps in services, knowledge, and cultural norms and promote women's rights through cross‐sector collaboration between health professionals, legal experts, policymakers, and community leaders.

## AUTHOR CONTRIBUTIONS

**Lokaratna Gyawali:** Data curation; investigation; resources; writing – review and editing. **Alok Atreya:** Conceptualization; supervision; writing – original draft; writing – review and editing. **Preza Kuinkel:** Writing – original draft; writing – review and editing. **Rabins Sanyal:** Writing – original draft; writing – review and editing. **Abhishek Shah:** Writing – original draft; writing – review and editing.

## FUNDING INFORMATION

No funding from an external source supported the publication of this case report.

## CONFLICT OF INTEREST STATEMENT

The authors declare that they have no conflict of interest regarding the publication of this case report.

## CONSENT STATEMENT

Written informed consent could not be obtained in this case due to the sensitive nature of the incident and ongoing legal proceedings. Identifying details have been removed to protect patient privacy. The investigating officer provided consent to publish this case report for academic purposes.

## Data Availability

The data presented in this case report are exclusively derived from a comprehensive autopsy, police inquest, and crime scene visits conducted in relation to the deceased infant. All essential data and information essential for a comprehensive understanding of the case have been meticulously documented within this manuscript. In consideration of the sensitive nature of this case and out of respect for privacy and legal constraints, we regretfully do not have any additional datasets or raw data available for sharing.
